# Novel Strategy for the Development of Antibacterial TiO_2_ Thin Film onto Polymer Substrate at Room Temperature

**DOI:** 10.3390/nano11061493

**Published:** 2021-06-04

**Authors:** Patcharaporn Phuinthiang, Dang Trung Tri Trinh, Duangdao Channei, Khakhanang Ratananikom, Sirikasem Sirilak, Wilawan Khanitchaidecha, Auppatham Nakaruk

**Affiliations:** 1 Department of Civil Engineering, Faculty of Engineering, Naresuan University, Phitsanulok 65000, Thailand; ryeoploy09@gmail.com (P.P.); wilawank1@gmail.com (W.K.); 2 Centre of Excellence for Innovation and Technology for Water Treatment, Faculty of Engineering, Naresuan University, Phitsanulok 65000, Thailand; tttdang247@gmail.com; 3 Department of Chemistry, Faculty of Science, Naresuan University, Phitsanulok 65000, Thailand; duangdaoc@nu.ac.th; 4 Department of Science and Mathematics, Faculty of Science and Health Technology, Kalasin University, Kalasin 46230, Thailand; khakhanang_r@yahoo.com; 5 Department of Community Medicine, Faculty of Medicine, Naresuan University, Phitsanulok 65000, Thailand; 6 Department of Industrial Engineering, Faculty of Engineering, Naresuan University, Phitsanulok 65000, Thailand

**Keywords:** titanium dioxide, antibacterial coating, disinfection surface, *E. coli*, *S. typhimurium*

## Abstract

This work demonstrates a novel method to deposit an antibacterial TiO_2_ thin film on a polymer substrate at room temperature. A combination of sol–gel and photon assistance was used in the experiment in order to avoid any thermal processes of thin film crystallization. The morphological photograph of samples indicated that the TiO_2_ thin film was perfectly coated on the PVC substrate without any cracks or pinholes. Chemical analysis by EDS and XPS reported that the thin film consisted of titanium (Ti), oxygen (O), and carbon (C). The Raman spectrum proved that the thin film was the anatase phase of TiO_2_ and, furthermore, that it was contaminated with carbon remaining from the photon assistance process. In addition, the optical band gap of the thin film was 3.35 eV, suggesting that the photocatalytic activity of TiO_2_ should occur under UV-A radiation. The bacteria viability assay was examined using *E. coli* and *S. typhimurium* as indicator strains under UV-A irradiation (365 nm) at different times. The data from OD and CFU count revealed that >97% of bacteria were killed after 60 min of irradiation, and the bacteria were completely killed at 120 min for *E. coli* and 180 min for *S. typhimurium*.

## 1. Introduction

At present, access to clean and reliable water resources, necessary for the activities and public health of human beings, has been reduced significantly due to the transmission of waterborne diseases through various substances, such as bacteria, viruses, and hazardous and radioactive materials [1,2]. Among microorganisms, *Escherichia coli* (*E. coli*) and *Salmonella typhimurium* (*S. typhimurium*) are leading pathogenic microorganisms which can cause dangerous diseases, such as diarrhea or gastroenteritis. It has been estimated that there are approximately 23 million cases of infection by *S. typhimurium* in Southeast Asia (SEA), with an incidence of around 4000 per 100,000 people/year [3]. Therefore, the prevalence of these pathogenic microorganisms in daily consumer products (e.g., drinking water and food) is a notable concern for public health professionals. According to water quality guidelines, drinking water is usually disinfected by the addition of chlorine. However, other mutagenic and carcinogenic disinfection by-products can be generated due to the reaction of chloride with natural organic compounds in the water. Furthermore, organisms also increase their own resistance to chlorine, which leads to higher concentrations being required for disinfection [4].

Alternatively, disinfection processes have been developed, including ozonation, ultraviolet (UV) irradiation, and advanced oxidation processes (AOPs). Among AOPs, photocatalytic processes using TiO_2_ (titania) can decompose not only organic pollutants but also inactive pathogenic microorganisms. The advantages of using TiO_2_ as a photocatalyst include its high degradation efficiency, low cost, and non-toxicity [5]. Several state-of-the-art studies have reported the efficiency of TiO_2_ photocatalysis to inactivate *E. coli* and *S. typhimurium* [6,7,8,9,10,11,12]. However, the drawbacks of this process are the separation of the photocatalyst from the suspension and the aggregation of suspended particles [13]. Therefore, the immobilization of TiO_2_ photocatalyst on a supporting material is an ideal method to solve these drawbacks. Several works have revealed that TiO_2_ can be coated on various supports with high performance, such as activated carbon [14], glass [15], metal foams [16], silica beads [17], and polymers [18,19]. However, the coating of TiO_2_ thin films on polymer substrates has not attracted much attention because the sintering of TiO_2_ requires a high temperature of ≥300 °C (~300 °C for brookite, 400–700 °C for anatase, and ≥600 °C for rutile), which degrades the polymer substrate. Although some coating processes, such as sputtering, can operate at a low temperature, these techniques require high-tech equipment [20], or use epoxy resin as a cover [21,22,23].

Therefore, the aims of the present work were to (1) develop a low-cost and simple coating process to coat TiO_2_ on the polymer substrate at room temperature by using sol–gel combined with photon-assistance techniques, and (2) to evaluate the photo-killing performance of the synthesized TiO_2_ thin film over *E. coli* under UV-A irradiation. The physical and chemical properties of the thin film were also characterized by scanning electron microscopy (SEM) coupled with energy dispersive X-ray analysis (EDX), X-ray photoelectron spectroscopy (XPS), Raman spectroscopy, and UV-Vis spectrophotometry.

## 2. Methodology

### 2.1. TiO_2_ Thin Film Coating

**Figure 1:**
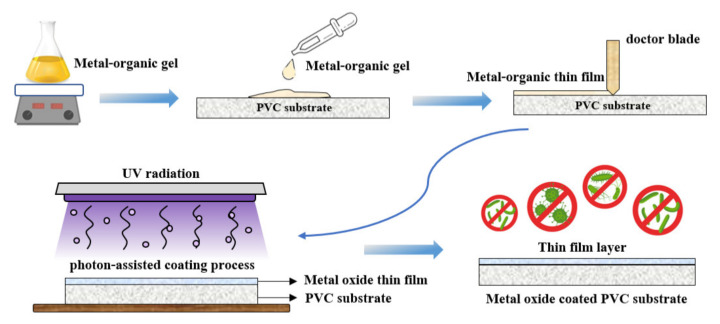
Schematic diagram of TiO_2_ coating process using a photon-assisted method.

A polyvinyl chloride (PVC) sheet was purchased from a local supplier in Thailand and used as a polymer substrate for this work. A metal–organic gel was prepared by mixing titanium isopropoxide (TTIP; Ti(OC_3_H_7_)_4_, 97%, Sigma-Aldrich) with acetic acid (C_2_H_4_O_2_, ≥99.5%, Sigma-Aldrich) and deionized (DI) water. The mixed solution was stirred at 60 °C for 10 h. Afterwards, the metal–organic gel was coated on the PVC by the doctor blade method. Finally, the coated sample was irradiated by UV-C 36 W (TUV 36W, Philips, Poland) for 3 h at room temperature (~25 °C) with ambient conditions. The summarization of the coating process is shown in Figure 1.

### 2.2. Thin Film Characterizations

Surface morphology and elemental composition of TiO_2_-coated PVC were analyzed using a scanning electron microscope (SEM, Leo1455VP, Carl Zeiss, Germany) coupled with energy dispersive X-ray analysis (EDX). X-ray photoelectron spectroscopy (XPS, Axis Ultra DLD, Kratos Analytical, UK) was used to examine the oxidation states and the surface composition as a function of binding energy. The crystal structure of the film was evaluated by a laser Raman spectrophotometer (LabRAM HR Evolution, HORIBA Scientific, Japan). The optical transparency and band gap of the film were obtained using a double-beam UV-Vis spectrophotometer (UV-6100 Spectrophotometer, Mapada, China).

### 2.3. Bacteria Viability Assay

A bacteria viability assay of the TiO_2_ thin film was carried out against *E. coli*, ATCC 25,922 and *S. typhimurium*, TISTR 1469 as indicator strains. Briefly, a single colony of bacteria was grown in nutrient broth for 16–18 h at 37 °C to obtain a bacterial concentration of 1 × 10^9^ CFU/mL. One hundred microliter of bacterial culture was plated on 2 cm (width) × 2 cm (length) coated and uncoated samples. These samples were activated under 10 W of UV-A (365 nm, single wavelength) for 0, 30, 60, 120, and 180 min. They were subsequently washed out with 10 mL nutrient broth to obtain a bacterial culture. Bacterial cultures were grown for 16–18 h at 37 °C to determine cell growth by monitoring OD 600 nm. Serial dilutions were prepared with nutrient broth; afterwards, 0.1 mL of bacterial cultures were spread onto nutrient agar plates. Colony counts were performed after 18 h incubation at 37 °C. The bacteria viability was determined by plotting visible counts (CFU/mL) against incubation time under UV-A radiation.

## 3. Results and Discussion

### 3.1. Physicochemical Characteristics of PVC and TiO_2_-Coated PVC

**Figure 2:**
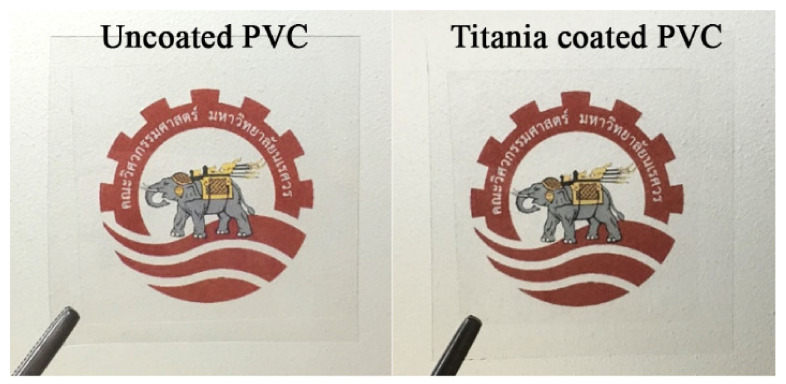
Photo of uncoated PVC and TiO_2_-coated PVC samples.

Figure 2 shows the appearance of coated and uncoated PVC on the Faculty of Engineering, Naresuan University logo. Both coated and uncoated PVC had very high transference, and no difference between the samples was observed. Since light diffraction did not occur on the coated sample, it can be said that the TiO_2_ film was very thin and smooth. Further, the TiO_2_ thin film was homogenous without any single cracks or pinholes. The coating process was conducted at room temperature; there was sufficient time for slowly removing hydrocarbon from the metal–organic gel and transforming it to the metal oxide thin film without creating any cracks or shrinkage.

**Figure 3:**
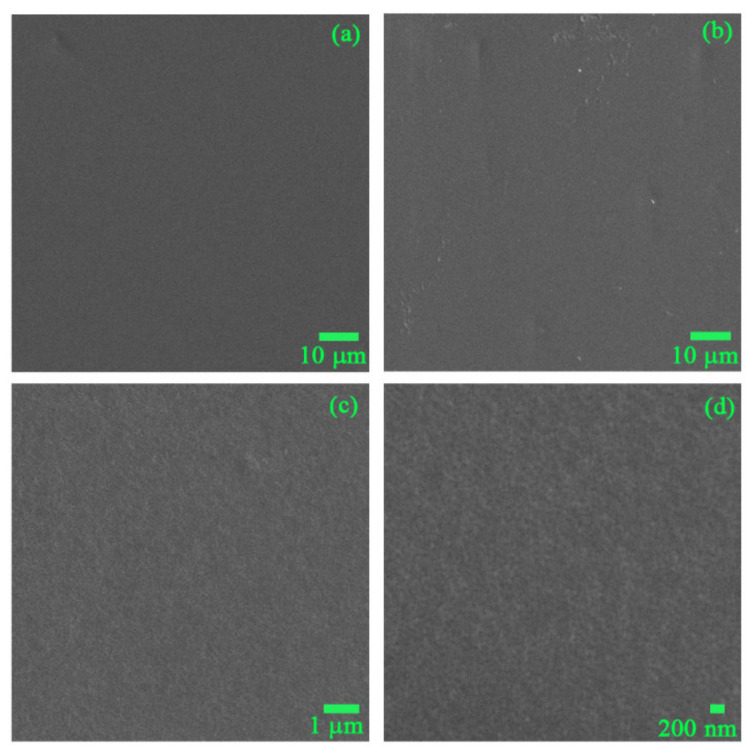
SEM images of (**a**) uncoated PVC, and TiO_2_-coated PVC at different magnifications: (**b**) ×500, (**c**) ×5000, and (**d**) ×10,000.

The surface morphology of the TiO_2_ thin film at different magnifications is shown in Figure 3. The thin film was perfectly formed without any cracks or pinholes, and the very small grain size of the TiO_2_ thin film was observed at high magnification (×10,000). The EDX data revealed that the main components of the thin film included oxygen (O, 46.13 at%), carbon (C, 41.68 at%), and titanium (Ti, 12.19 at%). The ratio of Ti/O was approximately ¼, representing the TiO_2_ bonding in the TiO_2_ thin film layer. However, the high amount of carbon (C) is likely derived from the PVC substrate, because the electron beam of EDX can penetrate to a depth of 1–2 µm.

**Figure 4:**
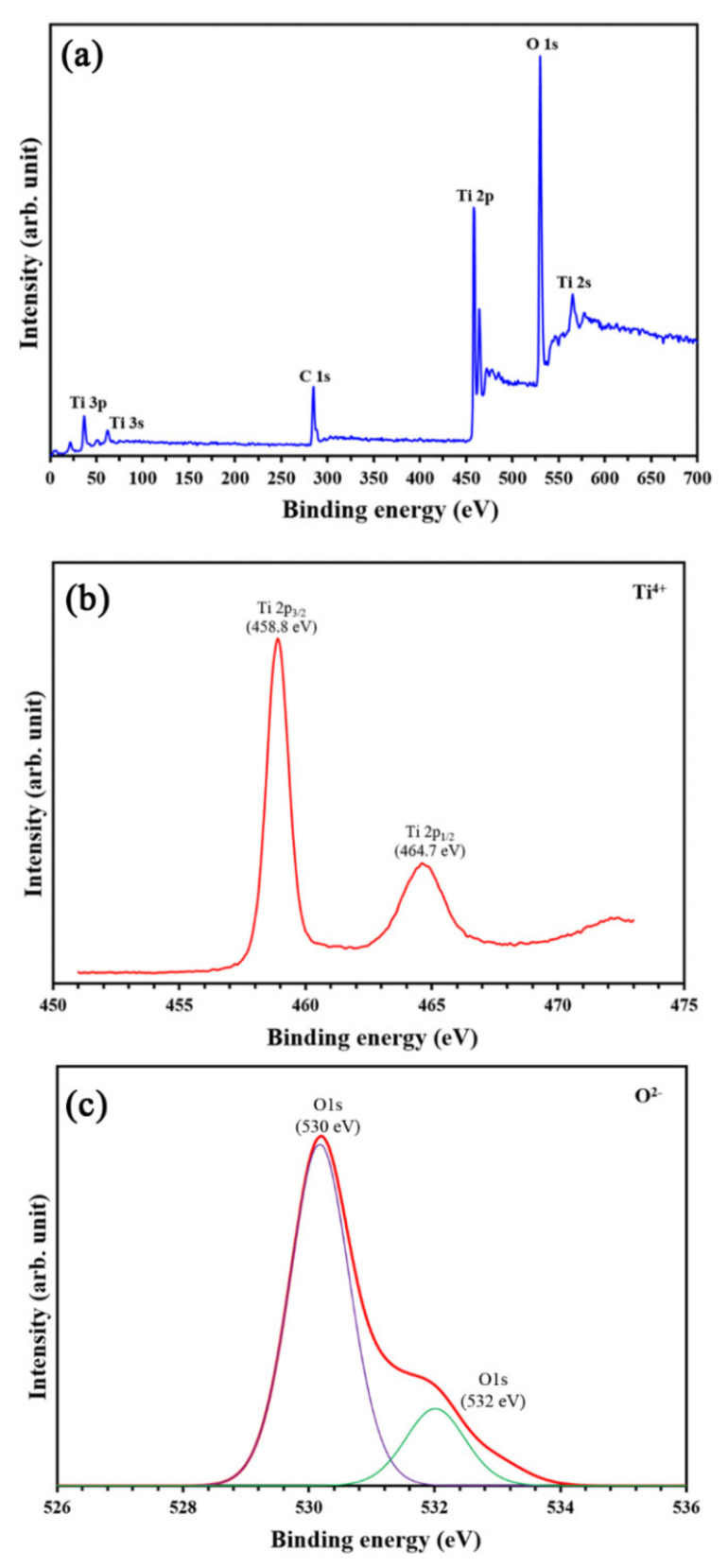
XPS spectra of (**a**) survey spectra, (**b**) spectra of the Ti2p, and (**c**) spectra of the O1s peaks of TiO_2_ thin film coated on PVC substrate.

The stoichiometry of the TiO_2_ thin film and the chemical state of the Ti atom were characterized using XPS analysis, as presented in Figure 4. The survey spectra of the coated PVC contained Ti2p and O1s peaks of TiO_2_. The XPS spectra of Ti2p were fitted with the doublet Ti2p_3/2_ (binding energy 458.8 eV) and Ti2p_1/2_ (binding energy 464.7 eV). The spin-orbit doublets of the two peaks were consistent with Ti^4+^ ions being the primary valence state in the TiO_2_ compound. In addition, Figure 4c shows the O1s core-level XPS spectra of the TiO_2_ film coated on the PVC sample.

The O1s signal revealed two peak positions at 530 and 532 eV. The main peak at ~530 eV could be ascribed to lattice oxygen in TiO_2_, while the signal at ~532 eV could be associated with surface hydroxyl groups. Regarding the aforementioned EDX and XPS results, the TiO_2_ was certainly formed as a thin film on the PVC substrate. However, the high intensity of the C1s peak referred to a large amount of existing carbon, which was from either the PVC substrate or the TiO_2_ thin film.

**Figure 5:**
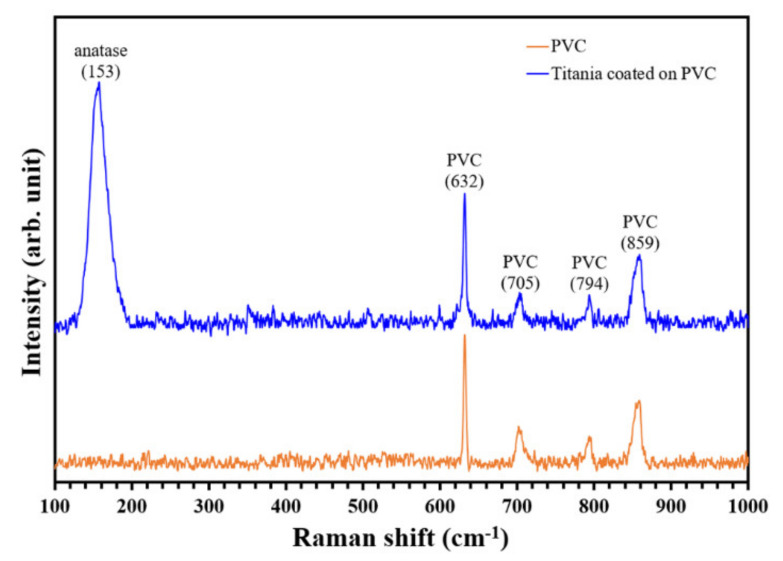
Quantification of phase content in TiO_2_ thin film by Raman spectra.

The phase content of the TiO_2_ thin film was analyzed by Raman analysis, and the result is presented in Figure 5. Strong Raman peaks at ~630, 700, 790, and 860 cm^−1^ were observed, representing the PVC substrate. However, the coated PVC obtained a strong peak at ~153 cm^−1^, referring to the anatase phase of TiO_2_. Theoretically, the main peak of anatase is located at ~143 cm^−1^, but the peak in this work was shifted to ~153 cm^−1^. This phenomenon can occur when the anatase phase is doped with cation atoms (i.e., carbon (C)) [24]. In this work, the EDX and XPS data revealed the presence of C atoms in the thin film, and this C impurity led to the peak shift in the Raman spectra.

**Figure 6:**
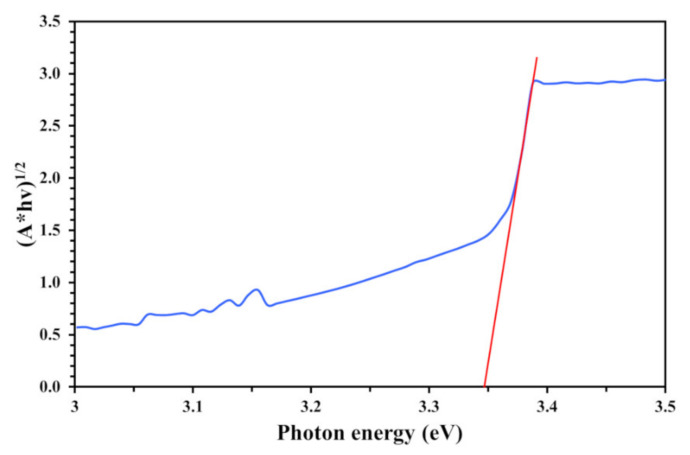
Tauc plot for TiO_2_-coated PVC substrate.

**Figure 7:**
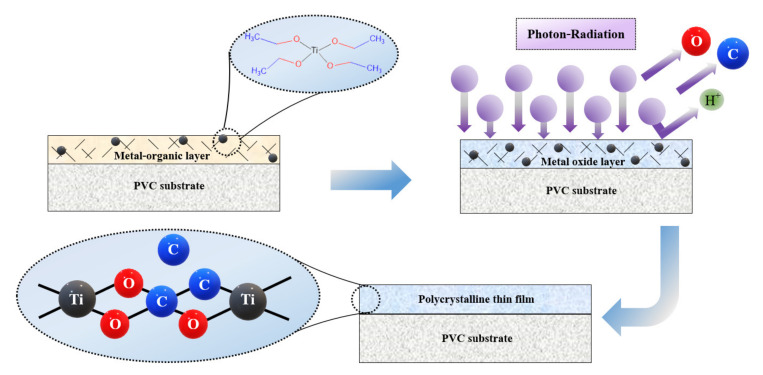
Summarization of the TiO_2_ film growth mechanism.

The optical band gap (Eg) of the thin film was analyzed using a Tauc plot [25]. The band gap of the titania thin film was estimated by drawing the interception of a plotted spectrum of (Ahυ)^1/2^ as a function of photon energy in the range of 3.0–3.5 eV, as shown in Figure 6. Extrapolation gave a band gap of ~3.34 eV for the TiO_2_-coated PVC substrate prepared by a novel photon-assisted coating process. Its wide band gap of 3.34 eV corresponds to a 370 nm photon wavelength (λ), which necessarily involves the use of UV-A light to achieve photoactivation. In the present work, the optical band gap of the synthesized TiO_2_ was 3.34 eV due to carbon contamination from the substrate and/or during the synthesis process. This ion contamination can cause three types of lattice defect: (1) cation replacement, (2) anion replacement, and (3) interstitial, as shown in Figure 7. These defects are known to change the crystallite size and lead to an increase in the optical band gap.

In the present work, carbon contamination in the TiO_2_ thin film led to a larger optical band gap than pure anatase. This may be due either to charged defects or to charged defects that were formed and neutralized by other defects. Hence, the blue shift in the band gap value by C-doping (as impurity) suggests an increase in the n-type carrier concentration; most of the C ions must be incorporated as interstitial donors into the structure rather than for the substitution of acceptors. The results in this work agree with other literature which suggests that the tuning of the band gap energy of metal oxide nanoparticles increases with metal ion doping [26]. Ahamed et al. reported that the band gap energy of TiO_2_ nanoparticles increased from 3.31 to 3.87 eV with increasing Zn dopant concentration [26].

A simple schematic illustration of the formation mechanism of the TiO_2_ thin film on the PVC substrate is presented in Figure 7. The metal–organic compound in the coating layer was deposited on the PVC substrate and cured through UV-C irradiation at room temperature, so the PVC substrate was subjected to only low levels of thermal stress. Under photon irradiation at room temperature, carbon and hydrogen elements were slowly removed from the metal–organic gel. As the curing times increased, the assistance of photons provided enough energy to transform amorphous compositions of Ti–OCH bonds to polycrystalline Ti–O bonds, known as the crystallization process. Finally, a perfect TiO_2_ thin film was formed on the surface of the PVC substrate without any cracks, shrinkage, or pinholes. However, the crystallization process could not occur perfectly without using any thermal energy. Hence, some of the C atoms from the metal–organic gel remained in the thin film layer, resulting in the changes in the physical and chemical properties of TiO_2_ discussed above.

### 3.2. Bacteria Viability Assay

**Figure 8:**
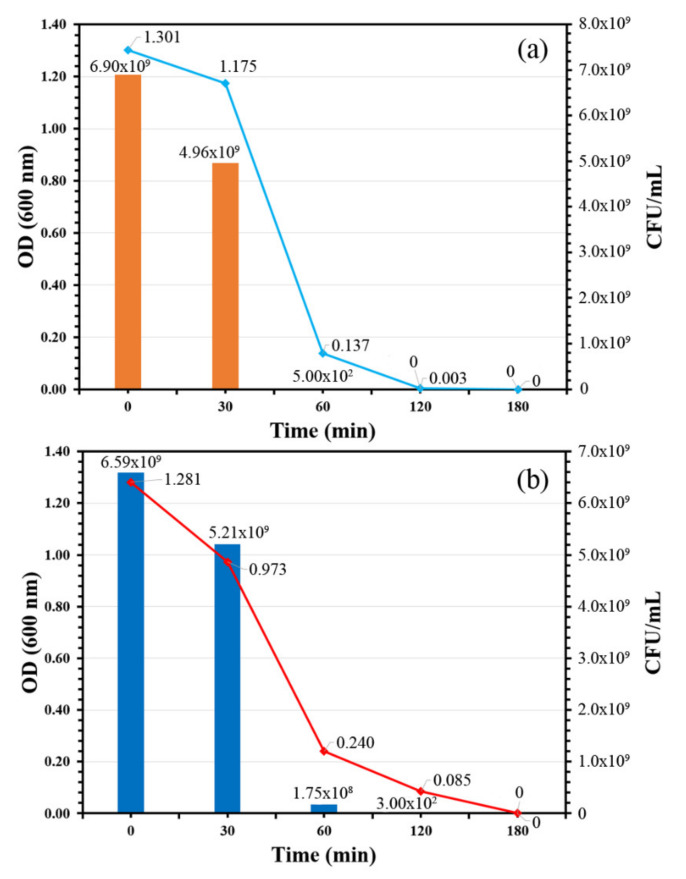
Time-killing curves of TiO_2_-coated PVC: (**a**) *E. coli* and (**b**) *S. typhimurium*.

**Figure 9:**
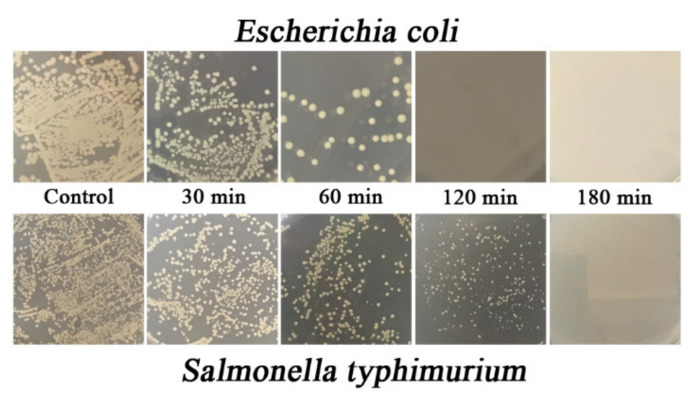
Photographs of agar plate manifestation of *E. coli* (**top**) and *S. typhimurium* (**bottom**) under different irradiation times.

A bacteria viability assay was used in this study to classify the antibacterial activity of TiO_2_-coated PVC and the relationship between antibacterial activity and illumination time. Two Gram-negative bacteria *E. coli* and *S. typhimurium* were chosen for the assay because of their problematic significance in global health worldwide. Figure 8 shows the time-killing curve of the TiO_2_ thin film against *E. coli* and *S. typhimurium*. In addition, photographs of agar plates of both bacteria under various irradiation times are shown in Figure 9.

The time-killing curve of the TiO_2_-coated PVC revealed that the bacteria count of both indicator strains in terms of CFU/mL and OD 600 nm decreased over time, indicating the antibacterial activity of the TiO_2_-coated PVC. In the case of *E. coli*, the bacteria count decreased from 6.90 × 10^9^ to 500 × 10^2^ CFU/mL within 60 min; in other words, more than 99% of the *E. coli* population was killed. The *E. coli* was completely killed after 120 min, as its bacteria count became zero. These results suggest that TiO_2_-coated PVC not only possesses antibacterial activity, but also has bactericidal properties against *E. coli*. A similar bactericidal pattern was found when *S. typhimurium* was used as an indicator strain. Approximately 97% of the *S. typhimurium* population was killed after 60 min of irradiation; the initial bacteria concentration was 6.59 × 10^9^ CFU/mL and decreased to 1.75 × 10^8^ CFU/mL. A count of zero CFU/mL was found after 180 min of irradiation time, indicating that *S. typhimurium* was completely destroyed. This clearly confirms the bactericidal property of TiO_2_-coated PVC.

The results in this work correlate well with those of Tsuang et al. [27], who found that TiO_2_ nanoparticles completely killed *E. coli*, *Pseudomonas aeruginosa*, *Staphylococcus aureus*, *Enterococcus hires*, and *Bacteroides fragilis* after 50 min of UV illumination. Moreover, their results elucidated that the UV dosage used for illumination did not affect the viability of bacteria, and all types of bacteria survived well in the absence of TiO_2_ nanoparticles. A bactericidal mechanism of TiO_2_ was proposed and explained by Sunada et al. [28]. The bactericidal mechanism consists of two steps: a relatively lower-rate photo-killing step and a higher-rate photo-killing step. The relatively lower-rate photo-killing step is the partial decomposition of the outer membrane of bacteria by strong reactive oxygen species (ROS) (i.e., h^+^, ·OH, O^2^^−^, H_2_O_2_) produced by TiO_2_. The bactericidal mechanism of TiO_2_ was further proposed by Othman et al. (2014). The authors explain that the TiO_2_ nanoparticle is involved in photophysical and photochemical processes. In a photophysical process, light excites the electrons of TiO_2_ to generate energized electron–hole pairs, which either recombine and dissipate the energy as heat or dissociate because of charge trapping, resulting in charge carriers for redox reactions in the photochemical process. In the photochemical process, the photoexcited electron–hole pairs participate with the adsorbed electron donors and absorbed electron acceptors such as water and oxygen on the TiO_2_ surface to produce highly reactive hydroxy radicals (OH^−^) and superoxide ions (O^2−^). These two reactive oxygen species are very powerful oxidants that can be used to oxidize and decompose organic matter, including bacteria. This process does not significantly influence cell death, but changes cell permeability, which allows ROS to easily access the cell membrane. Thus, ROS attach to the cell membrane, which can accelerate peroxidation of the polyunsaturated phospholipid component of the cell membrane, induce the loss of respiration activity and the leakage of cell components, and eventually cause cell death.

Notably, the complete elimination of bacteria might be observed at different irradiation times when using different types of indicator strains because of the difference in the structure of the bacterial cell envelope. Bacteria cell envelopes consist of two layers: cell membrane (the inside) and cell wall (the outside). Bacterial cell membranes are lipid semipermeable membranes which do not differ much between Gram-positive and Gram-negative bacteria. In contrast, the bacteria cell walls of Gram-negative and Gram-positive bacteria differ because of the thickness of the peptidoglycan layer and the presence or absence of the outer lipid membrane. Gram-positive bacteria have a thick peptidoglycan layer and no outer lipid membrane, while Gram-negative bacteria have a thin peptidoglycan layer and an outer lipid membrane. The outer membrane is believed to play an important role as a barrier to preventing ROS in the initial step of the bactericidal mechanism (the relatively lower-rate photo-killing step). The outer membrane is the first barrier, and once it is damaged the cytoplasmic membrane is attacked, leading to a loss of cellular respiration and eventually cell death. The authors of [28] explain that the photo-killing reaction for intact cells involves two steps: an initial lower-rate photo-killing step followed by a higher-rate one, as mentioned earlier. However, the reaction of spheroplasts, cells without cell walls, exhibited only the higher-rate photo-killing step, suggesting that the bacterial cell wall acted as a barrier to the photo-killing process. Therefore, different illumination times might be observed as a result of the different bacteria strains used.

## 4. Conclusions

This work succeeded in synthesizing a TiO_2_ thin film coated on a PVC substrate at room temperature using a combination of sol–gel and photon-assistance techniques. Due to the simplicity and low cost of this technique, TiO_2_ thin film coating can be performed without using thermal processes, high-tech equipment, or vacuum systems. In addition, various types of substrates can be applied due to the room temperature operation, and it is applicable for industrial applications. The analytical data revealed that the TiO_2_ thin film was perfectly coated on the PVC substrate as TiO_2_ (anatase phase, contaminated with carbon) with 3.35 eV optical band gap. Bacteria viability assay confirmed the excellent antibacterial ability of TiO_2_-coated PVC under UV-A (365 nm) irradiation: >99% for *E. coli* and >97% for *S. typhimurium* after 60 min. In addition, the bacteria were completely killed at 120 and 180 min for *E. coli* and *S. typhimurium*, respectively. The TiO_2_ coated on a polymer substrate as a thin film layer in this work could be used as an antibacterial packaging material for daily consumer products. Only when exposed to sunlight can excellent antibacterial ability be achieved through the photocatalytic process of the TiO_2_ thin film.
